# High-Throughput Sequencing Reveals the Regulatory Networks of Transcriptome and Small RNAs During the Defense Against *Marssonina brunnea* in Poplar

**DOI:** 10.3389/fpls.2021.719549

**Published:** 2021-09-08

**Authors:** Yangwenke Liao, Qingyue Zhang, Rongrong Cui, Xin Xu, Fuyuan Zhu, Qiang Cheng, Xiaogang Li

**Affiliations:** Co-Innovation Center for Sustainable Forestry in Southern China, College of Biology and the Environment, Nanjing Forestry University, Nanjing, China

**Keywords:** poplar, microRNA, transcriptome, regulatory networks, plant-fungi interaction, *Marssonina brunnea*

## Abstract

MicroRNAs are implicated in the adjustment of gene expression in plant response to biotic stresses. However, the regulatory networks of transcriptome and miRNAs are still poorly understood. In the present study, we ascertained the induction of genes for small RNA biosynthesis in poplar defense to a hemibiotrophic fungus *Marssonina brunnea* and afterward investigated the molecular regulatory networks by performing comprehensive sequencing analysis of mRNAs and small RNAs in *M. brunnea*-inoculated leaves. Differentially expressed genes in *M. brunnea*-infected poplar are mainly involved in secondary metabolisms, phytohormone pathways, the recognition of pathogens, and MAPK pathway in the plant, with real-time quantitative PCR (qPCR) validating the mRNA-seq results. Furthermore, differentially expressed miRNAs, such as MIR167_1-6, MIR167_1-12, MIR171_2-3, MIR395-13, MIR396-3, MIR396-16, MIR398-8, and MIR477-6, were identified. Through psRobot and TargetFinder programs, MIR167-1-6, MIR395-13, MIR396-3, MIR396-16, and MIR398-8 were annotated to modulate the expression of genes implicated in transportation, signaling, and biological responses of phytohormones and activation of antioxidants for plant immunity. Besides, validated differentially expressed genes involved in lignin generation, which were *phenylalanine ammonia-lyase, ferulate-5-hydroxylase, cinnamyl alcohol dehydrogenase*, and *peroxidase 11*, were selected as targets for the identification of novel miRNAs. Correspondingly, novel miRNAs, such as Novel MIR8567, Novel MIR3228, Novel MIR5913, and Novel MIR6493, were identified using the Mireap online program, which functions in the transcriptional regulation of lignin biosynthesis for poplar anti-fungal response. The present study underlines the roles of miRNAs in the regulation of transcriptome in the anti-fungal response of poplar and provides a new idea for molecular breeding of woody plants.

## Introduction

The plants confront variable biotic stresses on account of their immobility, such as the challenge of viruses, actinomyces, bacteria, fungi, nematodes, and pests (Chisholm et al., 2006; Eyles et al., 2010; Cagirici et al., 2017). During the response to pathogen infection, the plant can initiate the recognition of pathogen-associated molecular patterns (PAMPs)-triggered immunity and then induce alternations in proteins, protein kinases, and transcription factors, leading to the regulations of a great number of functional genes (Torres et al., 2006; Sturrock et al., 2011; Bilir et al., 2019). Simultaneously, plant own nucleotide-binding domain and leucine-rich repeat-containing proteins (NB-LRRs), which specialize in perceiving pathogen effectors and booting up effector-triggered immunity, resulting in hypersensitive reactions and activation of *R* genes (Torres et al., 2006). Hence, the expression of quantitative genes altered in plant cells in response to pathogen infection, while exploring the relationships between these genes and regulation of them is still challenging.

MicroRNAs (miRNAs) 21-24 nucleotides (nt) in size, which are small endogenous non-coding single-stranded RNAs synthesized by an RNA silencing system (Bhogireddy et al., 2021), play momentous roles in modulating the gene expression in the plant at the post-transcriptional level (Waterhouse and Hellens, 2015). An increasing number of reports have demonstrated that miRNAs regulate the growth, development, and stress responses in the plant by binding reverse complementary sequences and afterward bringing about the cleavage and/or translational inhibition of target mRNAs (Brant and Budak, 2018). The previous studies showed a bunch of differentially expressed miRNAs in plant species, such as cotton, cucumber, and tomato, during the interaction with pathogens, *via* high-throughput sequencing of small RNAs (Jin et al., 2012; Wang et al., 2018; Bilir et al., 2019). However, the regulatory mechanisms of miRNAs-adjusted genes during plant-pathogen interaction remain unclear.

Poplar (*Populus* sp.), an important forestry species growing fast and possessing high biomass, has become a model organism for tree research in recent years because of its broad distribution, genotypic diversity, and suitability for molecular analysis (Jansson and Douglas, 2007). The black spot disease caused by a hemibiotrophic fungus *Marssonina brunnea*, resulting in early defoliation, is a common poplar disease (Han et al., 2000). *M. brunnea* f. sp. *monogermtubi* can compatibly interact with certain plants from the *Leuce* section in the *Populus* genus and *M. brunnea* f. sp. *multigermtubi* can compatibly interact with some species from the *Aigeiros* section, which are two different specialized strains of *M. brunnea* identified in China (Zhang et al., 2018). Only several miRNAs have been mined involved in poplar defense to fungi pathogens (Chen et al., 2012; Chen and Cao, 2015; Su et al., 2018). In the present study, we performed high-throughput sequencing to analyze the changes in transcriptome profiles and small RNAs in *M. brunnea*-inoculated poplar leaves *in vitro*. In the current study, the experiments uncovered the regulatory networks of transcriptome and miRNAs in poplar defense to fungal infection and provided deeper insight into the interaction between trees and fungal pathogen.

## Materials and Methods

### Plant Material and Fungus

Stem cuttings of Nanlin895 (*Populus deltoides* × *Populus euramericana* “Nanlin 895”), part resistant to *M. brunnea* f. sp. *multigermtubi* strain (Chen et al., 2015), were subcultured *in vitro* to sprout, in Murashige and Skoog (MS) medium supplemented with naphthalene acetic acid (NAA, 0.4 mg L^−1^), 6-benzylaminopurine (6-BA, 4.0 mg L^−1^), thidiazuron (TDZ, 0.04 mg L^−1^), sucrose (25 g L^−1^), and agar (5.5 g L^−1^); and then transferred to MS medium added with NAA (0.2 mg L^−1^), 6-BA (4.0 mg L^−1^), sucrose (25 g L^−1^), and agar (5.5 g L^−1^), to grow into seedlings with 2–3 cm height. The culture condition was as followed: day/night temperature of 23/23°C, a 16/8 h light/dark period, and illumination intensity of 150 μmol m^−2^ s^−1^. Poplar seedlings subsequently grew in sterile nutrient soil-vermiculite (3:1) mix under day/night temperature of 26/20°C, with a 16/8 h light/dark period, and a 68–73% relative humidity. Pots were put in plastic trays used for sub-irrigation, and plants were supplied with water one time per week. Then, 7–10 days later, leaves were detached for inoculation experiments.

The fungus *M. brunnea* f. sp. *multigermtubi* strain was offered by Dr. Qiang Cheng and Dr. Qin Xiong (Nanjing Forestry University) and preserved on potato dextrose agar medium (PDA) at 4°C using slant tubes, which was isolated according to Cheng et al. (2010) from *P. canadensis* in the campus of Nanjing Forestry University and identified morphologically and molecularly according to Xiong et al. (2019). We smeared the preserved *M. brunnea* on PDA in a sterile Petri dish and then maintained the dishes at 25–28°C in the dark for 20 days until conidia elution and inoculation.

### Inoculation of Poplar Leaves With Fungus and Sample Collection

We eluted the conidia incubated on PDA with sterile ddH_2_O and adjusted their density to 6 × 10^6^ conidia L^−1^ for the inoculum, referring to the method of Liao et al. (2020). Afterward, we collected fully expanded healthy poplar leaves from seedlings, put them on a moist filter paper in Petri dishes, and dropped the spore suspension (30 μL) onto the abaxial leaf surface. The Petri dishes containing inoculated leaves were sealed and incubated in the dark at 25–28°C and relative humidity of 100%, and the disease symptoms on leaves were surveilled from 0 to 18 days postinoculation (dpi) with *M. brunnea*. As a negative control, mock inoculations were performed using sterile ddH_2_O alone.

The inoculated leaves were harvested at 3, 6, 12, 24, and 48 h post inoculation (hpi) to analyze the expression of genes related to the miRNA biosynthesis, at 6 and 24 hpi to sequence mRNAs and small RNAs, and at 3, 6, 10, and 18 dpi to examine the disease symptoms of *M. brunnea* on poplar leaf. One biological sample was obtained by pooling three to four leaves, and three biological replicates were analyzed for each treatment. The experiments were independently performed four times.

### Detection of Fungal Infection

*Marssonina brunnea* infection status was evaluated at 3, 6, 10, and 18 dpi by aniline staining and microscopy, according to the study of Liao et al. (2020). Briefly, the inoculated leaf section was cut into 1 × 1 cm piece, immersed in 0.15% saturated trichloroacetic acid bleaching liquid (solvent, 3:1, alcohol:chloroform solution) for 12 h for decoloration, and then stained in a mixed solution (saturated chloral hydrate and aniline blue, 5:1) at 58°C for 4 h. The stained leaf pieces were detected and photographed using a light microscope (BH200, SDPTOP, Sunny Instruments, Ningbo, China).

### RNA Extraction and mRNA-Seq Analysis

The total RNA of leaf samples pooled at 6 and 24 hpi was extracted using HiPure HP Plant RNA Mini Kit (Magen, Guangzhou, China) for mRNA-seq analysis, as described in the instructions from the manufacturer. Next, mRNA was purified by the interaction of the poly (A) tails and magnetic oligo (dT) beads, and double-stranded cDNA was synthesized to construct the library. RNA sequencing services were provided by Personal Biotechnology Co., Ltd. (Shanghai, China). The cDNA libraries were examined by an Agilent High Sensitivity DNA Kit on Agilent 2100 Bioanalyzer (Agilent, St. Clara, CA, USA) with an average fragment length of 200–300 bp. The libraries were sequenced by Illumina NextSeq500 (Illumina Inc., San Diego, CA, USA) to generate paired-end reads with 150 bp length.

The transcriptome was analyzed following the procedure of Guo et al. (2017). The raw data were transformed into FASTQ format by the software of the sequencing platform. To get high-quality clean data, connectors and low-quality reads were filtered by Cutadapt (v1.16). Clean reads were used for mapping analysis by Bowtie2 (2.2.6) and Tophat2 (2.0.14), and the reference genome for gene annotation was *P. deltoides* WV94 V2.1 (https://phytozome-next.jgi.doe.gov/). HTSeq (0.9.1) and DESeq (1.30.0) were used for gene expression analysis at different expression levels (Supplementary Table 2). The *R* package, edgeR, was used to identify the differentially expressed genes (DEGs) (Robinson et al., 2010). The expression level of each unigene was calculated and normalized to generate FPKM. In the present study, the selection of DEGs was based on |log_2_ fold change| ≥ 1 with *p*-value < 0.05. The threshold of the *p*-value was determined using a false discovery rate (FDR) in multiple tests.

### Small RNA-Seq and Bioinformatics Analysis

The same RNA samples used for the mRNA-seq were used for the miRNA-seq. Small RNA libraries were constructed using NEB Next Multiplex Small RNA Library Prep Kit (New England Biolabs, Inc., Hitchin, UK), following the instructions of the manufacturer on an Illumina NextSeq500. The ligation of total RNA (10 μg) from 12 independent samples belonging to 4 groups, respectively, with 3′ adapters and 5′ adapters was performed using the T4 RNA ligase. Double-stranded cDNA was synthesized *via* RNA reverse-transcription using Superscript II Reverse Transcriptase. DNA fragments, which were enriched by PCR, were segregated by polyacrylamide electrophoresis (PAGE) gel. Next, the verification of fragment size and DNA library distribution was performed using Agilent 2100 for quality control of fragments enriched by PCR. After detecting the total concentration by Picogreen, the constructed libraries were single-end sequenced on the HiSeq 2500 platform at Personal Biotechnology Co., Ltd. (Shanghai, China).

The number of clean reads, with sequence lengths more than 18 nt and less than 36 nt, was counted, which were applied for small RNA analysis. The identical sequence in one single sample was deduplicated and the calculation of sequence abundance was carried out to obtain the unique reads, which were subsequently compared with the Rfam (14.0) database by BLAST. Four types of known non-coding RNAs (rRNA, tRNA, snRNA, and snoRNA) were screened, with a screening criterion of 0–2 mismatches. Unique reads which were not annotated to the above four types of non-coding RNAs were compared to mature miRNA sequences of the known miRNA in miRBase22 by BLAST, with screening criteria of 0–2 mismatches. Unique reads, not aligned with the Rfam and miBase databases, were compared to the genome to predict the novel miRNAs through the Mireap online program (http://sourceforge.net/projects/mireap/), using the default criteria of the program. Their secondary structure maps were drawn by RNA fold. Inter-sample correction of total reads was required for the standardization of expression amount, and then, the gene expression pattern of the sample was comprehensively investigated by count per million (CPM). The DEM (differentially expressed miRNA) selection was based on |log2 fold change| ≥ 1 and *p*-value < 0.05 using DESeq. Finally, sequences for target genes of DEMs were predicted by psRobot_tar and TargetFinder (Bo and Wang, 2005; Zhao et al., 2020). The analysis script of these two programs was shown in Supplementary Table 7. Target genes that met the criteria of both programs were sorted out for the subsequent discussion.

### Real-Time Quantitative PCR Assay

For the real-time quantitative PCR (qPCR) of miRNA biosynthesis-associated gene expression in poplar response to *M. brunnea* and the validation of RNA-seq results, total RNA of leaves sampled at different time points was extracted using a plant RNA Extraction Kit (Tiangen, Beijing, China) and reverse-transcribed using a ReverTra Ace qPCR RT Kit (Toyobo, Osaka, Japan). Sequences of genes associated with miRNA biosynthesis and for verification of mRNA sequencing were mined from *P. deltoides* WV94 V2.1 (https://phytozome-next.jgi.doe.gov/). All primers used were shown in Supplementary Table 6. The qPCR was performed using the SYBR Green PCR MasterMix (Vazyme, Nanjing, China), and the cycling conditions were as follows: 95°C for 5 min, followed by 40 cycles of denaturation at 95°C for 15 s, and annealing at 60°C for 30 s. Fluorescent signals were collected during the 60°C step. To validate the amplification of a single product, a melt curve, with conditions as 95°C for 15 s, 60°C for 1 min, and 95°C for 15 s, was generated at the end of the PCR cycles using software offered by the Step One Real-Time PCR Detection System (ThermoFisher, Waltham, MA, USA). The cDNA used for the validation of small RNA-seq was reverse-transcribed from 1 μg of total RNA for each sample using a miR-X miRNA First-Strand Synthesis Kit (Takara, Beijing, China), and the qPCR analysis was performed on the CFX Connect Real-Time PCR Detection System (Bio-Rad, Hercules, CA, USA) at Personal Biotechnology Co., Ltd. (Shanghai, China), using the TB Green Premix Ex TaqII (Takara, Beijing, China). The *actin* gene and 5S were used as internal genes. The threshold cycle (Ct) value of the internal gene was subtracted from that of the gene of interest to obtain a ΔCt value. The Ct value of the mock-inoculated control sample was subtracted from the ΔCt value to obtain a ΔΔCt value. The fold changes in expression level relative to the control were expressed as 2^−ΔΔCt^ (Livak and Schmittgen, 2001).

## Results

### Phenotype After *M. brunnea* Inoculation and miRNA Biosynthetic Gene Expression in Local Poplar Leaf

**Figure 1:**
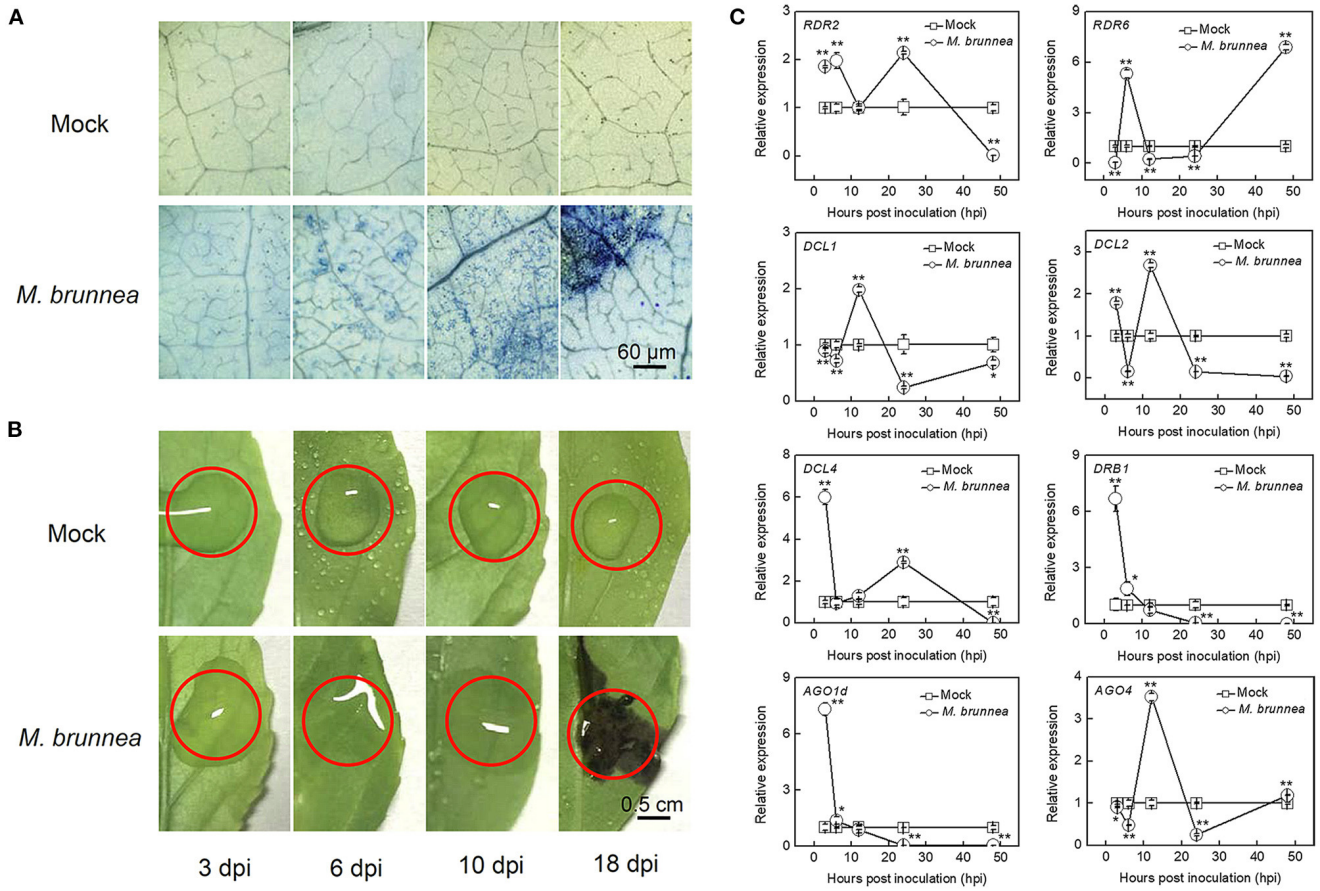
Disease symptoms and gene expression of poplar leaves at different time points post *Marssonina brunnea* inoculation. **(A)** Light micrographs of leaf pieces stained with aniline blue and **(B)** photographs at 3, 6, 10, and 18 days postinoculation (dpi). Area within red cycle in **(B)** is inoculated; bar = 60 μm in **(A)** or 0.5 cm in **(B)**. **(C)** The expression of microRNA biosynthetic genes at 3, 6, 12, 24, and 48 h postinoculation (hpi). Leaf samples were collected from the inoculated leaves with *M. brunnea* and mock inoculations were performed with sterile ddH_2_O alone as a negative control. The square indicates mock treatment and the cycle represents *M. brunnea* inoculation, with positive and negative error bars. The results are presented as mean values ± SD; *n* = 3. Asterisks represent significant differences in gene expression between treatments of mock and *M. brunnea* inoculation, according to Duncan's test. *P < 0.05; **P < 0.01. RDR, RNA-dependent RNA polymerase; DCL, dicer-like protein; DRB, double-stranded RNA-binding protein; AGO, argonate protein.

We first inoculated the abaxial surface of Nanlin895 leaf with *M. brunnea* and monitored the disease-developing process. Aniline blue staining revealed that *M. brunnea* successfully infected poplar, with secondary hyphae, emerged 3 dpi and then spread, which then significantly grew and developed to the whole leaf at 18 dpi (Figure 1A). Further, disease symptoms were photographed at corresponding time points. No distinct phenotype appeared at 3 dpi. The black spots merged and formed a dark brown imprint at 18 dpi (Figure 1B). This suggested that *M. brunnea* can infect Nanlin895 plants and compatibly interacts with this species.

To estimate the initiation of miRNA expression and determine appropriate sampling time points for mRNA and miRNA expression profiling, we tested the dynamic responses of critical genes involved in small RNA biosynthesis at 3, 6, 12, 24, and 48 hpi (Figure 1C). *RDR2* and *RDR6* encoding RNA-dependent RNA polymerases, play central roles in 24-nt-long repeat-associated small RNAs (rasiRNAs) and small interfering RNAs (siRNAs) biosynthesis; *DCL1* encoding Dicer-like (DCL) enzymes is critical in processing miRNAs, and *AGO1d* encoding the Argonaute (AGO) protein is responsible for the RNA-induced silencing complex (RISC) formation, which regulates expression of target mRNAs in aspects of plant biology; *DCL4* and *DCL2* function in producing siRNAs; *AGO4* serves in the loading of rasiRNAs; and *DRB1* encoding dsRNA-binding protein is a cofactor of DCL (Bhogireddy et al., 2021). All the above genes were significantly triggered by *M. brunnea* inoculation before 48 hpi. Compared with mock, the transcript level of *RDR2* increased to 1.85-, 1.98-, and 2.14-fold, respectively, at 3, 6, and 24 hpi, and *RDR6* expression was, respectively, upregulated to 5.30- and 6.88-fold at 6 and 48 hpi; the transcript level of *DCL1* increased to 1.98-fold at 12 hpi and the expression of *DCL2* elevated to 1.78- and 2.67-fold, respectively, at 3 and 12 hpi, while the transcript level of *DCL4* reached the peaks of 5.99- and 2.89-fold, respectively, at 3 and 24 hpi; *DRB1* showed apparent increases to 6.71-fold at 3 hpi; and *M. brunnea* inoculation strongly enhanced *AGO1d* transcription to 7.31-fold and *AGO4* to 3.52-fold, respectively, at 3 and 12 hpi (Figure 1C). These results indicate that *M. brunnea* infection can rapidly activate the RNA silencing system to synthesize small RNAs in poplar leaves before 24 hpi.

### Functional Genes Expression of *M. brunnea*-Inoculated Poplar Leaves Based on High-Throughput Sequencing

To examine the response of functional genes expression to *M. brunnea* infection, we performed high-throughput sequencing of mRNAs in poplar leaves at 6 and 24 hpi. After the removal of adapter sequences and low-quality reads, a total of 40893478/41398298/38410968, 39250688/41064414/39597552, 40176520/39938864/41793126, and 46429992/44517684/46841894 mRNA sequences were obtained for Mk_6h_1/2/3 (three replicates of mock treatment at 6 hpi), Mb_6h_1/2/3 (*M. brunnea* inoculation at 6 hpi), Mk_24h_1/2/3 (Mock at 24 hpi), and Mb_24h_1/2/3 (*M. brunnea* inoculation at 24 hpi), respectively. The proportion of clean reads mapped to the *P. deltoides* genome in each library was above 88.5%, and the proportion of uniquely mapped reads ranged from 91.41 to 92.35% (Supplementary Table 1), with normalized gene expression (FPKM) of all clean reads presented in Supplementary Table 2. A principal component analysis (PCA) was performed to compare the transcriptome characteristics of all samples intuitionally (Supplementary Figure 1). The samples from two-time points were significantly divided and the replicates were closely spaced, while the samples at 6 hpi were not isolated between mock and *M. brunnea* inoculation, indicating that more genes were differentially expressed at 24 hpi rather than 6 hpi with fungal pathogen.

Furthermore, we found 4,976 (2,396 up, 2,580 down) DEGs in the group of Mk_24h vs. Mk_6h (Mk_24h/Mk_6h), 996 (646 up, 350 down) DEGs in the Mb_6h/Mk_6h group, 3,936 (1,758 up, 2,178 down) DEGs in the Mb_24h/Mb_6h group, and 1,923 (561 up, 1,362 down) DEGs in the Mb_24h/Mk_24h group (Supplementary Figure 2A), with the clustering analysis of DEGs expression pattern in a heatmap (Supplementary Figure 2C). Despite the most (4,976) DEGs exhibited in Mk_24h/Mk_6h group, only 490 of them were differentially expressed in the Mb_6h/Mk_6h comparison and the rest were due to the sampling time point. Besides, 1,914 DEGs were overlapped in Mk_24h/Mk_6h and Mb_24h/Mb_6h comparisons, attributed to the time point (Supplementary Figure 2B). Both the Venn diagram and the heatmap showed intuitional time-specific expression patterns in this study. Further, there were 412 (41%) DEGs in Mb_6h/Mk_6h group excluded from Mk_24h/Mk_6h and Mb_24h/Mk_24h comparison and 1,073 (56%) DEGs in Mb_24h/Mk_24h group excluded from Mb_24h/Mb_6h and Mb_6h/Mk_6h comparison (Supplementary Figure 2B). These data certified the effect of *M. brunnea* inoculation on mRNAs and the reliability of transcriptome analysis in the present study.

**Figure 2:**
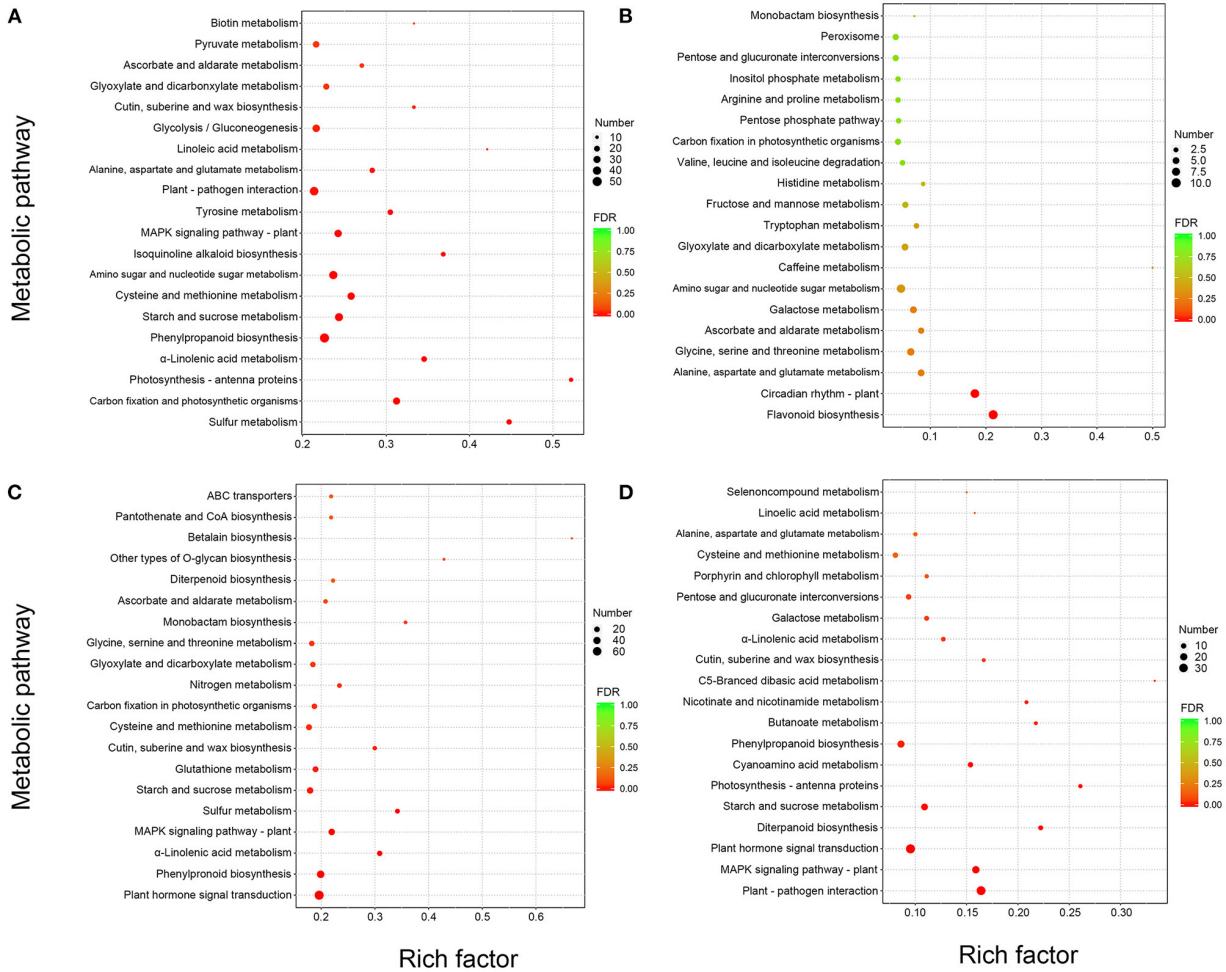
Enrichment analysis of all DEGs. The top 20 Kyoto Encyclopedia of Genes and Genomes (KEGG) pathways with the most significant enrichment of **(A)** Mk_24h/Mk_6h, **(B)** Mb_6h/Mk_6h, **(C)** Mb_24h/Mk_24h, and **(D)** Mb_24h/Mb_6h, accordingly. The “/” implies “vs.” The diameter of the cycle is positively correlated with the number of DEGs, and the color of the cycle indicates the false discovery rate (FDR) value as the scale shows.

To better study the highlighted pathway in response to *M. brunnea* infection in poplar, we performed a Kyoto Encyclopedia of Genes and Genomes (KEGG) annotation analysis for the DEGs. The top 20 enriched KEGG pathways of each comparison were showcased in Figure 2. Among the DEGs of Mk_24h/Mk_6h group, “Sulfur metabolism,” (pop00920) “Carbon fixation and photosynthetic organisms,” (pop00710) “Photosynthesis-antenna proteins,” (pop00196) and “α-Linolenic acid metabolism” (pop00592) ranked from the first to fourth, followed by “Phenylpropanoid biosynthesis,” (pop00940) “Starch and sucrose metabolism,” (pop00500) “Cysteine and methionine metabolism,” (pop00270) and “Amino sugar and nucleotide sugar metabolism.” (pop00520) (Figure 2A) Among the DEGs of Mb_6h/Mk_6h, “Flavonoid biosynthesis” (pop00941) ranked the first, followed by “Circadian rhythm,” (pop04712) “Alanine, aspartate, and glutamate metabolism,” (pop00250) “Glycine, serine, and threonine metabolism,” (pop00260) and “Ascorbate and aldarate metabolism.” (pop00053) “Tryptophan metabolism” (pop00380) was also apparently enriched in the Mb_6h/Mk_6h DEGs (Figure 2B). Among the DEGs of Mb_24h/Mk_24h, “Plant-pathogen interaction” (pop04626) was the most significantly changed pathway, followed by “MAPK signal pathway,” (pop04016) “Plant hormone signal transduction,” (pop04075) “Diterpenoid biosynthesis,” (pop00904) and “Starch and sucrose metabolism.” (pop00500) A significant alternation also emerged in “Phenylpropanoid biosynthesis” (pop00940) (Figure 2C). Besides, “Plant hormone signal transduction,” (pop04075) “Phenylpropanoid biosynthesis,” (pop00940) and “MAPK signaling pathway” (pop04016) ranked high among the Mb_24h/Mb_6h DEGs (Figure 2D). More inhibited genes appeared in these pathways than induced genes. Finally, 26 genes belonging to seven pathways involved in the poplar response to fungal attack were focused. The selected pathways were “Flavonoid biosynthesis,” (pop00941) “Phenylpropanoid biosynthesis,” (pop00940) “Tryptophan metabolism,” (pop00380) “Plant-pathogen interaction,” (pop04626) “MAPK signal pathway,” (pop04016) “Plant hormone signal transduction,” (pop04075) and “Diterpenoid biosynthesis,” (pop00904) with potential genes involved in poplar response to fungal infection as shown in Table 1.

**Table 1:** Potential genes may be involved in poplar response to *Marssonina brunnea*.

**Category**	**Gene ID**	**Gene description**	**Regulation**
Phenylpropanoid	Podel.19G001400	Shikimate O-hydroxycinnamoyltransferase (HCT)	↑
biosynthesis	Podel.10G229600	Phenylalanine ammonia-lyase (PAL)	↑
	Podel.07G018500	Ferulate-5-hydroxylase (CYP84A, F5H)	↑
	Podel.14G110800	Caffeic acid 3-O-methyltransferase (COMT)	↓
	Podel.16G069500	Cinnamyl alcohol dehydrogenase GroES-like domain (CAD)	↑
	Podel.10G135900	Peroxidase 11 (class III POD)	↑
	Podel.01G233500	β-glucosidase (bGL)	↓
Flavonoid	Podel.03G190100	Chalcone synthase (CHS)	↑
biosynthesis	Podel.15G053500	Leucoanthocyanidin reductase (LAR)	↑
	Podel.03G127400	Anthocyanidin synthase/ leucoanthocyanidin dioxygenase (ANS/LDOX)	↑
	Podel.04G144800	Flavonol synthase (FLS)	↓
Plant hormone	Podel.04G034400	Transport inhibitor response 1 (TIR1)	↑
signal transduction	Podel.06G269000	Auxin-responsive protein IAA 19 (IAA19)/ (Aux/IAA family)	↓
	Podel.01G127200	Small Auxin Up RNA family protein (SAUR)	↑
	Podel.14G021600	F-box protein GID2 (GID2, SLY1)	↑
	Podel.17G162900	DELLA protein	↓
	Podel.14G115200	Phytochrome-interacting factor 3 (PIF3)	↑
	Podel.01G177300	TIFY 10A, a JAZ domain-containing protein	↓
	Podel.02G196200	Basic/helix-loop-helix transcription factor 14 (bHLH14)	↑
Tryptophan metabolism	Podel.04G173600	Amidase (AMI)	↑
Diterpenoid biosynthesis	Podel.15G136600	Gibberellin-44 dioxygenase (GDOX)	↑
	Podel.04G116600	Cytochrome P450, family 82, subfamily G, polypeptide 1 (CYP82G1)	↑
Plant-pathogen	Podel.11G016000	RPM1-interacting protein 4 (RIN4)	↑
interaction	Podel.13G009000	Disease resistance protein resistance to *Pseudomonas syringae* 2 orthology, leucine-rich repeat-containing protein, RPS2	↑
MAPK signaling	Podel.09G036500	Protein phosphatase 2C 3-related	↑
pathway	Podel.05G267900	Catalase	↑

**Figure 3:**
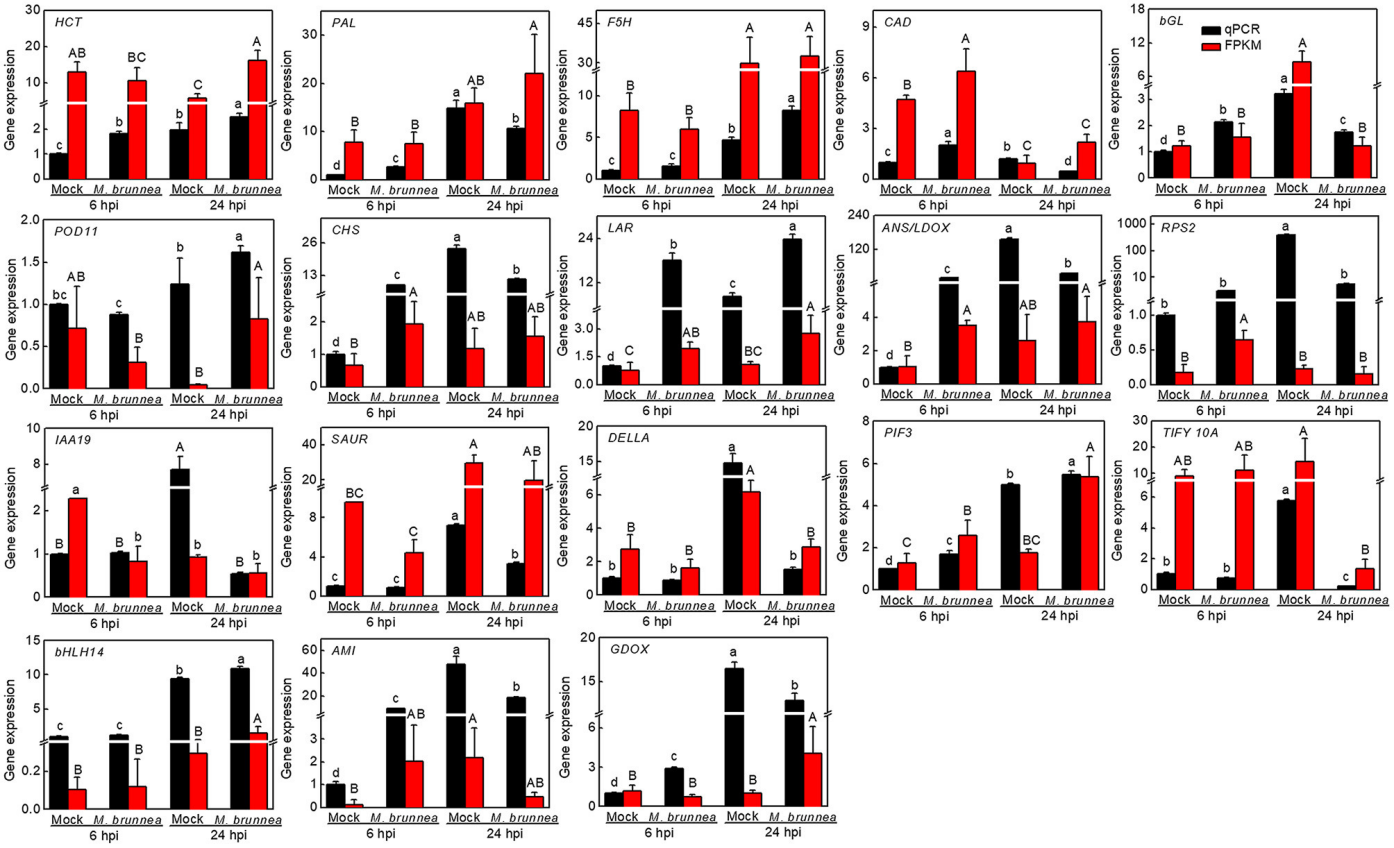
Quantitative PCR (qPCR) verifies the quality of transcriptome sequencing using the 2^−ΔΔCt^ method. The black bar represents the qPCR validation result of mRNA in the sample, with positive and negative error bars; and the red bar represents the normalized result (FPKM) of the sample in the transcriptome. The results of qPCR are presented as mean values ± SD; *n* = 3. The results of FPKM are presented as mean values; *n* = 3. Lowercase letters indicate significant differences in qPCR results between treatments, with capital letters for FPKM values (*P* < 0.05).

To validate the reliability of RNA-seq results, 18 genes were selected for qPCR validation, with data shown in Figure 3. The expression of genes involved in the phenylpropanoid pathway, such as *shikimate O-hydroxycinnamoyltransferase* (*HCT*, Podel.19G001400), *phenylalanine ammonia-lyase* (*PAL*, Podel.10G229600), *cinnamyl alcohol dehydrogenase-related* (*CAD*, Podel.16G069500), and β*-glucosidase* (*bGL*, Podel.01G233500), were significantly upregulated by *M. brunnea* inoculation at 6 hpi with the fungal pathogen, while the expression of *PAL, CAD*, and *bGL* decreased at 24 hpi. Expression of *ferulate-5-hydroxylase* (*F5H*, Podel.07G018500) and *peroxidase 11* (*POD11*, Podel.10G135900, and encoding class III POD) increased at 24 hpi (Almagro et al., 2009), together with *HCT* transcription. With respect to flavonoid biosynthetic genes, the abundance of *chalcone synthase* (*CHS*, Podel.03G190100), *leucoanthocyanidin reductase* (*LAR*, Podel.15G053500), and *anthocyanidin synthase*/*leucoanthocyanidin dioxygenase* (*ANS*/*LDOX*, Podel.03G127400) exhibited at a very high level at 6 h post *M. brunnea* inoculation. At 24 hpi, *CHS* and *ANS* were expressed to a lower level, with an obvious increase in *LAR* transcription. The *resistance to Pseudomonas syringae 2 orthology* (*RPS2*, Podel.13G009000), encoding an NB-LRR protein functioning in the recognition of pathogen, was slightly upregulated at 6 hpi but downregulated at 24 hpi. After the *M. brunnea* inoculation, *auxin-responsive protein IAA 19* (*IAA19*, Podel.06G269000) from Aux/IAA family, a repressor of auxin signaling, was inhibited at 24 hpi; and one gene from *Small Auxin Up RNA* family (*SAUR*, Podel.01G127200) mediating polar auxin transport was also downregulated. The transcript level of *DELLA* (Podel.17G162900) was reduced at 24 hpi, while *phytochrome-interacting factor 3* (*PIF3*, Podel.14G115200) was induced both at 6 and 24 hpi. The expression of *TIFY 10A* (Podel.01G177300) and the transcription factor *bHLH14* (Podel.02G196200) did not change at 6 hpi, while a dramatic decrease occurred in the former gene expression and an obvious increase emerged in the transcription of the latter gene at 24 hpi. Besides, *amidase* (*AMI*, Podel.04G173600) involved in tryptophan metabolism and free auxin production, and *gibberellin-44 dioxygenase* (*GDOX*, Podel.15G136600) functioning in gibberellin (GA) biosynthesis expressed to a higher extend at 6 hpi while to a lower level at 24 hpi. Therefore, the results of qPCR assays agreed with the transcriptome data (Figure 3). The qPCR results suggest that genes related to the biosynthesis of phenylpropanoid pathway-derived lignin were generally activated, with flavonoid biosynthetic genes strongly induced within 24 h post *M. brunnea* inoculation. Furthermore, the plant-fungi interaction and pathways of auxin, GA, and jasmonic acid (JA) were induced by a fungal infection.

### Annotations of Known miRNAs and Identification of Differentially Expressed miRNAs in *M. brunnea*-Inoculated Poplar Leaves

**Figure 4:**
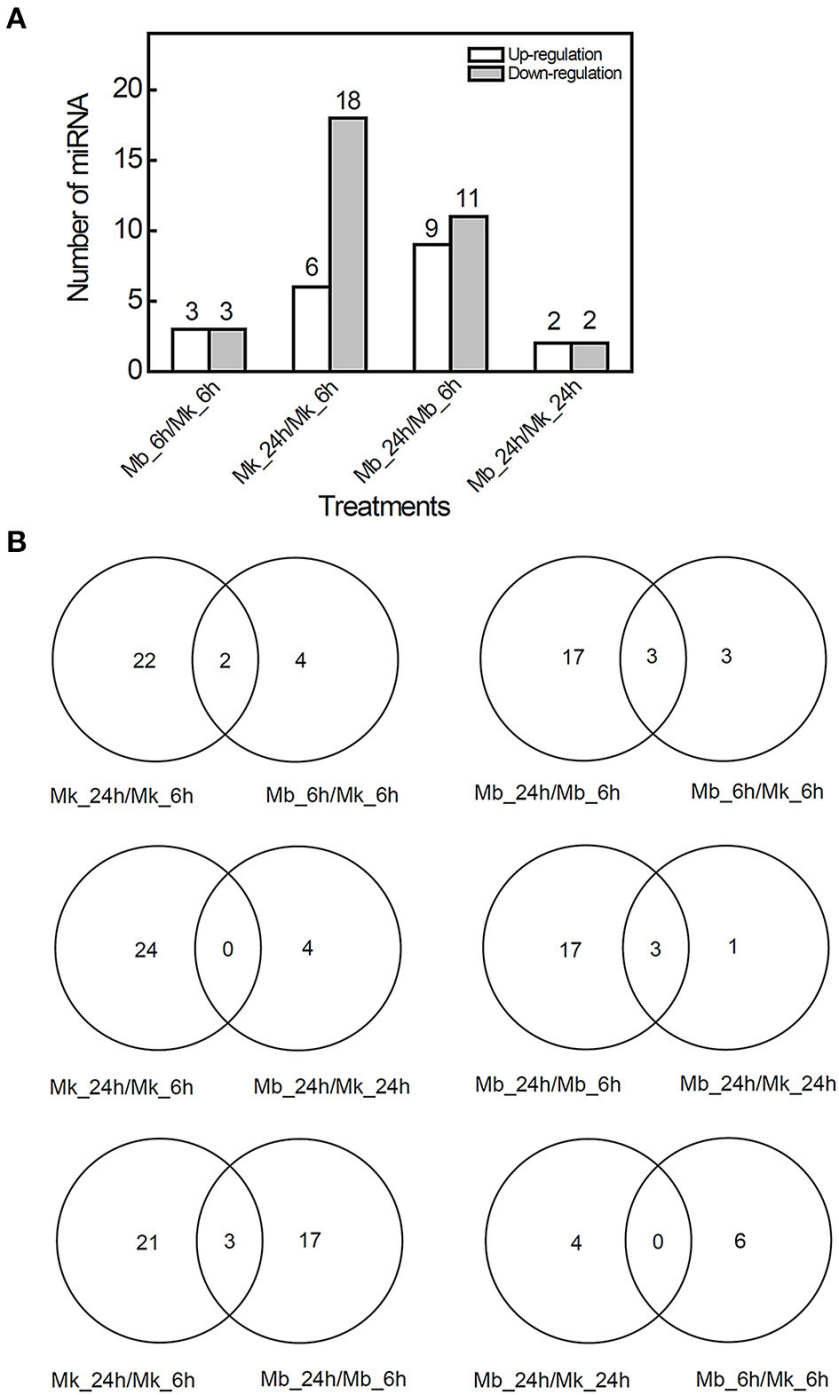
Analysis of differential known microRNA expression in *M. brunnea*-infected poplar leaves. **(A)** The upregulated and downregulated (|log_2_ foldchange| ≥1 and *P* <0.05) amount and **(B)** the Venn diagram of differentially expressed known miRNAs (DEMs).

Clean reads (≥18 nt) were produced by small RNA sequencing ranging from 11.59 to 26.05 million from 12 small RNA libraries (Supplementary Table 3). Most of the non-coding RNAs in total reads were ribosomal RNA (rRNA) while the unknown group contributed to the majority in unique reads (Supplementary Figure 3). Clean reads were BLAST searched against known mature miRNAs and pre-miRNAs of miRBase (version 22.0) to identify the known conserved miRNAs. According to the difference in expression (|fold change| > 2, *p*-value < 0.05) in the multi-sample group comparison, we found six known DEMs in the Mb_6h/Mk_6h group (3 miRNAs were upregulated and 3 miRNAs were downregulated), 24 DEMs in Mk_24h/Mk_6h comparison (6 miRNAs were upregulated and 18 miRNAs were downregulated), 20 DEMs in the Mb_24h/Mb_6h group (9 miRNAs were upregulated and 11 miRNAs were downregulated), and 4 DEMs in the Mb_24h/Mk_24h group (2 miRNAs were upregulated and 2 miRNAs were downregulated) (Figure 4). The Venn diagram showed that two DEMs were both included in Mk_24h/Mk_6h and Mb_6h/Mk_6h groups while the Mk_24h/Mk_6h and Mb_24h/Mk_24h comparisons did not share any DEM. Most of the DEMs in Mk_24h/Mk_6h can be attributed to the change of time point. In addition, only three DEMs were shared by Mk_24h/Mk_6h and Mb_24h/Mb_6h comparisons, and 10 DEMs of Mb_6h/Mk_6h group were different from that of Mb_24h/Mk_24h comparison, suggesting that *M. brunnea* inoculation caused diverse effect at 6 and 24 hpi on miRNAs in poplar leaves. The heatmap also presented intuitional time-specific expression patterns (Supplementary Figure 4).

The miRNAs complementarily paired bind target sites to regulate the transcription of target genes which we predicted using psRobot_tar and TargetFinder based on poplar genomic and transcriptomic databases. We selected the target genes that met the criteria of both programs.

Eight of these DEMs from Mb_6h/Mk_6h, Mb_24h/Mb_6h, and Mb_24h/Mk_24h groups (DEMs that we failed to predict target gene were excluded) and their target genes may be implicated in the poplar response to *M. brunnea* infection (Table 2), with a fold change of them shown in Supplementary Table 5.

**Table 2:** Potential known miRNAs, novel miRNAs, and the predicted targets may be involved in poplar response to *M. brunnea*.

**MicroRNA**	**Mature sequence**	**Regulation**	**Target ID**	**Target description**
Pde-MIR167_1-6	AGGTCATCTTGCAGCTTCAAT	↑	Podel.19G075500	SNARE associated Golgi protein family
Pde-MIR167_1-12	GGAAGCTGCCAGCATGATC	↑	Podel.18G116000	—
Pde-MIR171_2-3	TTGAGCCGTGCCAATATCACG	↑	Podel.16G149200	—
Pde-MIR395-13	GTTCCCTTGAGCACTTCA	↑	Podel.09G052200	ABC-2 type transporter family protein
Pde-MIR396-3	TTCCACAGCTTTCTTGAAC	↓	Podel.01G226500	Chaperone DnaJ-domain superfamily protein
			Podel.02G196400	GTP binding
			Podel.14G078200	Ribosomal RNA adenine dimethylase family protein
			Podel.T131700	Serine protease inhibitor, potato inhibitor I-type family protein
Pde-MIR396-16	TTCCACAGCTTTCTTGAACA	↓	Podel.02G196400	GTP binding
			Podel.T131700	Serine protease inhibitor, potato inhibitor I-type family protein
Pde-MIR398-8	GGAGCGACCTGGAATCACATG	↓	Podel.05G102600	Tudor/PWWP/MBT domain-containing protein
Pde-MIR477-6	ATCTCCCTCAAAGGCTTCCTC	↓	Podel.07G123500	—
Novel MIR3228	GCTGGGTTTATTTTTGAT	—	Podel.07G018500.1	Ferulate-5-hydroxylase (CYP84A, F5H)
Novel MIR8567	AGGGTATGGTCTGCATTGCTTTGA	—	Podel.10G229600.1	Phenylalanine ammonia-lyase (PAL)
Novel MIR5913	GATGCTGTGCCTCTGGCTAAT	—	Podel.16G069500.1	Cinnamyl alcohol dehydrogenase GroES-like domain (CAD)
Novel MIR6493	ACCGTCACACCCCAGAAGTG	—	Podel.10G135900.1	Peroxidase 11 (class III POD)

*Tables with white shading exhibit the known miRNAs, while gray showing novel miRNAs*.

Some of the selected DEMs targeted the same genes. For instance, both MIR396-3 and MIR396-16 engaged *GTP-binding* (Podel.02G196400) and *Serine protease inhibitor* (Podel.T131700) as targets. Besides, *Chaperone DnaJ-domain superfamily protein* (Podel.01G226500) and *Ribosomal RNA adenine dimethylase family protein* (Podel.14G078200) were also targeted by the same MIR396-3. *SNARE associated Golgi protein family* (Podel.19G075500), mainly involved in vesicle-associated membrane fusion, was targeted by MIR167_1-6. *ABC-2 type transporter family protein* (Podel.09G052200), primarily serving in plant hormones exportation, was the target of MIR395-13. *Tudor/PWWP/MBT domain-containing protein* (Podel.05G102600) was aimed by MIR398-8. However, Podel.18G116000, Podel.16G149200, and Podel.07G123500, which were target genes of MIR167_1-12, MIR171_2-3, and MIR477-6, respectively, were not annotated in the reference database.

### Identification of Novel miRNAs and Their Targets

**Figure 5:**
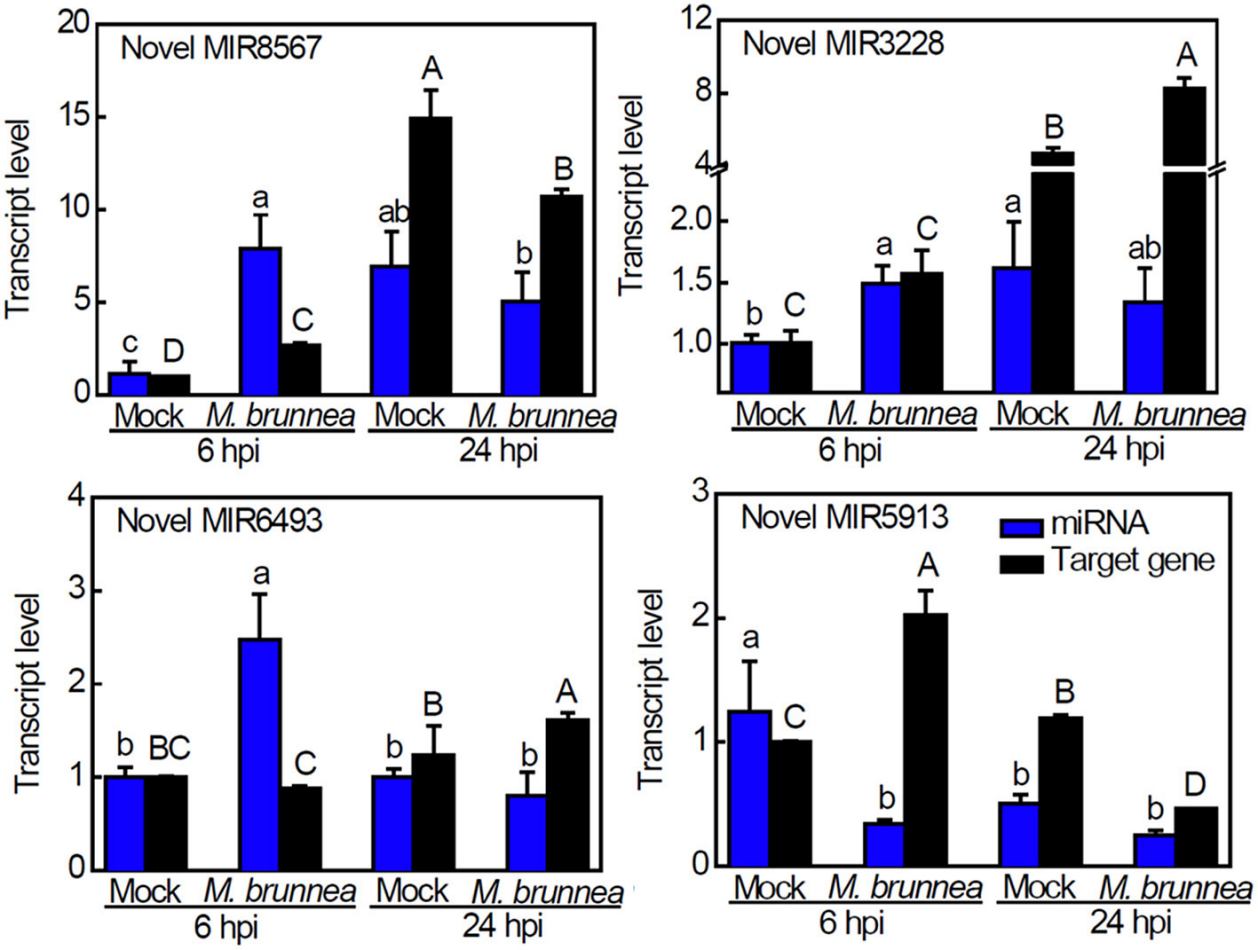
Expression analysis of candidate novel miRNAs and targets using the 2^−ΔΔCt^ method. Expression analysis of novel miRNA and the predicted target gene in the sample. The blue bar represents the qPCR validation result of novel miRNA and the black bar stands for the qPCR result for the target gene. The results of qPCR are presented as mean values ± SD; *n* = 3. The results of FPKM are presented as mean values; *n* = 3. Lowercase letters indicate significant differences in qPCR results between treatments, with capital letters for FPKM values or the qPCR-validated target gene expression (*P* < 0.05).

The MIREAP platform was hired to predict the precursors of new small miRNAs when the score was ≥ 2.2, with the RNAfold web server for a description of their secondary structures. Potential novel miRNAs were identified based on several important previously validated DEGs that we selected, and we identified four potential novel miRNAs (Table 2). The secondary structures predicted for the precursors of these candidate novel miRNAs are shown in Supplementary Figure 5, with red and blue asterisks indicating the 5′- and 3′-end positions of mature novel miRNA on the secondary structure of the precursor, respectively. Their expression patterns were analyzed along with their targets by qPCR verification (Figure 5). Compared to mock, the significant accumulation of Novel Pde-MIR8567 occurred at 6 hpi with no apparent change in abundance at 24 hpi, while the transcription of its target gene exhibited an increase at 6 hpi but decrease at 24 hpi. Novel Pde-MIR3228 expressed to a higher extent at 6 hpi but showed no obvious alternation at 24 hpi, and the target gene was only induced at 24 hpi. With respect to Novel Pde-MIR6493, an induction emerged at 6 hpi rather than 24 hpi, while its target expression showed an increase at 24 hpi but no significant change at 6 hpi, relative to the transcript levels in mock. At 6 hpi, the expression of Novel Pde-MIR5913 was inhibited and its target was upregulated, while no obvious change occurred to the transcript level of this novel miRNA. However, no cleavage among the novel miRNAs and their targets was validated by qPCR assays (Figure 5).

## Discussion

Evidence indicated that miRNAs can be crucial in transcriptional control of the fungi pathogen-responsive genes during the infection process in various species, such as cotton, tomato, and cucumber, using high-throughput sequencing and bioinformatics tools (Jin et al., 2012; Wang et al., 2018; Bilir et al., 2019). However, miRNA-mRNA regulatory networks during poplar response to pathogenic fungi infection remain unknown. In the present study, we found the rapid activation of miRNA biosynthesis in *M. brunnea*-infected poplar leaves, implying miRNA biosynthesis was initiated to respond to the fungal infection at the early stage. Therefore, we further performed transcriptome analysis of *M. brunnea*-inoculated poplar leaf at 6 and 24 hpi. The results showed that genes involved in phenylpropanoid biosynthesis, flavonoid biosynthesis, plant hormone signal transduction, diterpenoid biosynthesis, tryptophan metabolism, plant-pathogen interaction, and MAPK signaling pathway were significantly induced in poplar response to *M. brunnea* infection. We further selected 26 candidate genes from DEGs, and the qPCR results confirmed the data of transcriptome analysis.

Genes associated with the phenylpropanoid pathway responsible for producing the three lignin monomers called monolignols, such as *PAL* (Podel.10G229600) and *CAD* (Podel.16G069500), were induced by *M. brunnea* at 6 hpi, and *HCT* (Podel.19G001400), *F5H* (Podel.07G018500), and *POD11* (Podel.10G135900), were significantly upregulated at 24 hpi; and *bGL* (Podel.01G233500) consuming the intermediate cinnamic acid was downregulated at 24 hpi (Figure 3). Our results were supported by previous studies (Chen et al., 2020; von Tiedemann et al., 2021). Thus, lignin biosynthesis is intensively accelerated in the poplar defense against a fungal pathogen. Notably, *caffeic acid 3-O-methyltransferase* (*COMT*, Podel.14G110800) exhibited downregulation after fungal inoculation (Table 1 and Supplementary Table 2). HCT accounts for producing precursors of Guaiacyl and Sringyl unit lignin (Wagner et al., 2007), while COMT serves in the Sringyl unit lignin biosynthesis (Goujon et al., 2003). Poplar may prefer tamping cell wall with Guaiacyl unit lignin in defense to *M. brunnea*. Lignin formation can be initiated by the signal transduction of auxin and GA in plant immunity (Zhang et al., 2020). Accordingly, we found the enhancement of biosynthesis and signal transduction of auxin and GA (Figure 3). Although inhibition of auxin-responsive *SAUR* (Podel.01G127200) expression occurred, it may be attributed to the *AMI* (Podel.04G173600)-activated local auxin during the early responsive stage (Spartz et al., 2012).

The flavonoid biosynthesis branches at *p*-coumaroyl CoA from the phenylpropanoid pathway (Besseau et al., 2007). In the present study, *CHS* (Podel.03G190100), *LAR* (Podel.15G053500), and *ANS*/*LDOX* (Podel.03G127400) were explosively induced by fungal inoculation. Poplar may trigger flavonoids accumulation to generate antibiotic quinones, which further synthesizes polymeric compounds to form a protective barrier and scavenge reactive oxygen species to manage fungal invasion (Pourcel et al., 2007; Agati et al., 2012). On the other hand, the MAPK signal pathway was differentially expressed by *M. brunnea* infection in poplar, which also serves as a modifier of antioxidant systems (Xing et al., 2008; Meng and Zhang, 2013). Consistently, we found a higher transcript level of *protein phosphatase 2C* (Podel.09G036500) at 6 hpi, as well as *catalase* (Podel.05G267900) (Table 1). NB-LRRs can recognize specific effectors of pathogen and then activate MAPK cascade (Torres et al., 2006; Tsuda et al., 2013). In the present study, a slight increase in the expression of *RPS2* (Podel.13G009000) encoding an NB-LRR emerged at 6 hpi in poplar leaves. Taken together, fungal infection boots up a plant NB-LRR and MAPK pathway and simultaneously generates flavonoids, leading to the enhancement of antioxidant systems in poplar defense.

To further reveal the miRNA-mRNA regulatory networks during the poplar defense to pathogenic fungi, the small RNA-seq was performed at the same time points post inoculation. Among 43 DEMs, we listed eight DEMs from Mb_6h/Mk_6h, Mb_24h/Mk_24h, and Mb_24h/Mk_24h comparisons and predicted four novel miRNAs, according to the DEGs we selected (Table 2). MIR167 can accumulate to manage the auxin signaling pathway by manipulating its target *ARF factor* after bacterial pathogen infection (Jodder et al., 2017), supporting our result that *M. brunnea* inoculation led to upregulation of MIR167_1-6 and MIR167_1-12. The target gene of MIR167_1-6, Podel.19G075500, encodes a soluble *N*-ethyl-maleimide sensitive factor attachment adaptor protein receptor (SNARE) domain associated Golgi protein which is mainly involved in vesicle-associated membrane fusion. Recent research proved that QA-SNARE SYP132, a low-abundant, secretory SNARE localizing to the plasma membrane, is tightly modulated by auxin and that abundant SYP132 caused a restriction in auxin-derived apoplast acidification and vegetative growth (Xia et al., 2019). In the present study, poplar may augment MIR167_1-6 to repress SNARE, amplifying the auxin responses to fungal infection. Accordingly, the transcriptome analysis confirmed the enhancement of auxin biosynthesis and signaling (Table 1 and Figure 3).

MIR395 family is usually involved in stress-response processes. Augment in MIR395 led to suppression in WRKY26, thereby reducing the expression of some *PR* genes (Zhang et al., 2017). Our results exhibited that MIR395-13 accumulated after *M. brunnea* inoculation. The target gene *ABC-2 type transporter family protein* (Podel.09G052200) plays a key role in exporting phytohormones, such as salicylic acid (SA), jasmonic acid (JA), auxin, and strigolactone (Ye et al., 2013; Sasse et al., 2015; Dhara and Raichaudhuri, 2021). Increased MIR395-13 production might inhibit *ABC-2 type transporter* expression to block SA exportation, which thereby alleviated *PR* genes transcription in poplar response to pathogenic fungi (Dhara and Raichaudhuri, 2021). Furthermore, MIR396 can balance plant growth and disease resistance by interacting with growth-regulating factors in the plant. Blocking MIR396 expression resulted in higher grain yield while augment of MIR396 increased susceptibility to the blast fungus in rice (Chandran et al., 2019). In the present work, *M. brunnea* inoculation suppressed MIR396-3 and MIR396-16 expression. These two miRNAs coordinately regulate *GTP binding* (Podel.02G196400). During the early response to *M. brunnea*, abundant GTP-binding protein may rope JAZ domain-containing protein to provoke JA-signaling cascade in stomatal defense in poplar (Lee et al., 2018). Correspondingly, transcriptome analysis exposed the downregulation of the JA pathway inhibitor *TIFY 10A* (Podel.01G177300) expression by fungal infection (Table 1 and Figure 3). The previous study demonstrating that Nanlin 895 poplar restricted the SA pathway and activated the JA pathway in defense to *M. brunnea* infection supported the findings in this study (Liao et al., 2020).

MIR398 serves to store ${\text{O}}_{2}^{\cdot -}$ *via* inhibiting antioxidant enzymes activity in plants under stress (He et al., 2021). The production of target gene *Tudor/PWWP/MBT domain-containing protein* (Podel.05G102600) can interact with histidine-rich calcium-binding protein (Yang et al., 2020). The suppression of MIR398-8 in this study may manipulate Ca^2+^ signaling to amplify antioxidant systems and restrict reactive oxygen species accumulation.

**Figure 6:**
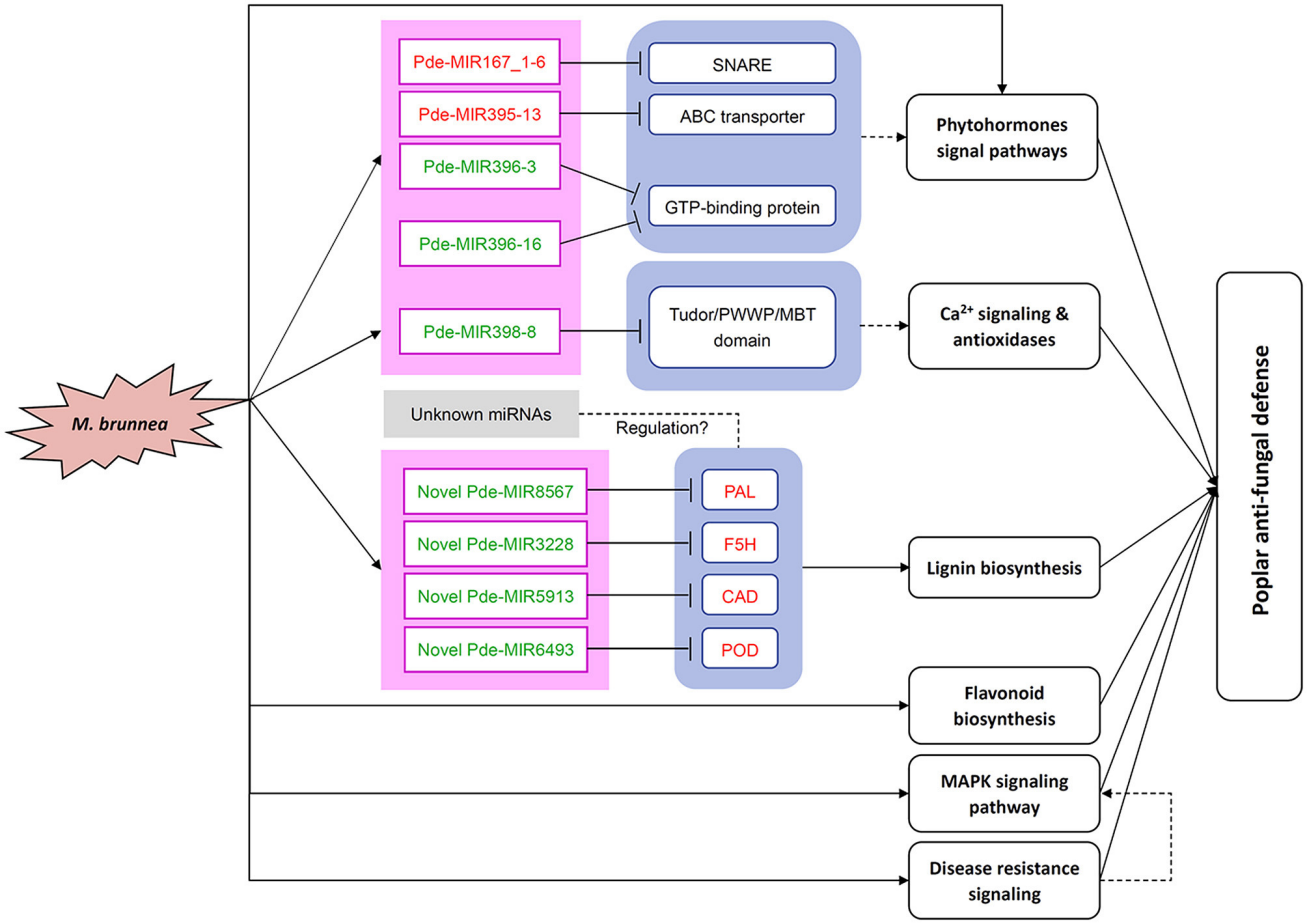
Overview of miRNAs and genes regulation patterns in the poplar response to *M. brunnea*. The red letters are upregulated genes or miRNAs or pathways, and the green letters are downregulated genes or miRNAs or pathways. “ → ” represents induction of mentioned elements or pathway, while “⊥” stands for suppression. A dashed line indicates speculated pathways. SNARE, soluble N-ethyl-maleimide sensitive factor attachment adaptor protein receptor; ABC transporter, ATP-binding cassette transporter; PAL, phenylalanine ammonia-lyase; CAD, cinnamyl alcohol dehydrogenase; POD, peroxidase; F5H, ferulate-5-hydroxylase; MAPK, mitogen-activated protein kinase.

Noteworthily, four novel miRNAs were predicted, including Novel MIR8567, Novel MIR3228, Novel MIR5913, and Novel MIR6493 target to the upregulated lignin biosynthetic DEGs which are *PAL, F5H, CAD*, and *POD11*, respectively. All these target genes are critical genes to lignin biosynthesis (Figure 6). However, qPCR assays did not validate the cleavage among the novel miRNAs and their targets, implying the probable existence of other unknown miRNAs regulating the target genes above. Overall, miRNA-mRNA regulatory networks play pivotal roles in defense against fungal attacks in poplar. However, the detailed interactions and roles of the novel miRNAs were predicted and other unknown miRNAs in poplar defense to pathogen await further research.

## Conclusion

In summary, global transcriptional profiles of mRNA and small RNAs were investigated in *M. brunnea*-infected poplar leaves, combined with qPCR validation. We obtained numerous DEGs and selected 26 critical fungal pathogen-responsive genes. The functional analysis demonstrated the involvement of these DEGs in lignin and flavonoid biosynthesis, phytohormone pathways, disease resistance signaling, and MAPK pathway. Further, we screen out DEMs and identified eight annotated miRNAs, four novel miRNAs, and their targets involved in poplar response to *M. brunnea* infection. By analyzing the function of the targets, we found that these miRNAs mediated plant hormones pathways, antioxidant systems enhancement, and lignin biosynthesis. According to the above analysis, we summarized these regulatory networks in Figure 6. The results of our research underline the role of miRNAs in poplar defense to fungal infection and provide a new idea for the molecular breeding of trees.

## Data Availability Statement

The datasets presented in this study can be found in online repositories. The names of the repository/repositories and accession number(s) can be found at: https://www.ncbi.nlm.nih.gov/, PRJNA730976; https://www.ncbi.nlm.nih.gov/, PRJNA731035

## Author Contributions

YL and XL designed experiments. QZ, RC, and XX performed experiments. YL, QZ, RC, and FZ analyzed data. YL and XL wrote and revised the article. All authors contributed to the article and approved the submitted version.

## Funding

This work was supported by the National Natural Science Foundation of China project (Grant No. 31600482) and the Natural Science Foundation of Jiangsu Province (Grant No. BK20190040). This work was supported by the Co-Innovation Center for Sustainable Forestry in Southern China of the College of Biology and Environment at Nanjing Forestry University, Nanjing, China.

## Conflict of Interest

The authors declare that the research was conducted in the absence of any commercial or financial relationships that could be construed as a potential conflict of interest.

## Publisher's Note

All claims expressed in this article are solely those of the authors and do not necessarily represent those of their affiliated organizations, or those of the publisher, the editors and the reviewers. Any product that may be evaluated in this article, or claim that may be made by its manufacturer, is not guaranteed or endorsed by the publisher.
