# Supplementary material for: High-Throughput Sequencing Reveals the Regulatory Networks of Transcriptome and Small RNAs During the Defense Against Marssonina brunnea in Poplar
Source: Front Plant Sci. 2021 Sep 8;12:719549. doi: 10.3389/fpls.2021.719549 (PMC8456019; doi:10.3389/fpls.2021.719549)
Supplement: Supplementary file 8 [file Data_Sheet_1.PDF]

Supplementary Material

1. Supplementary Figures

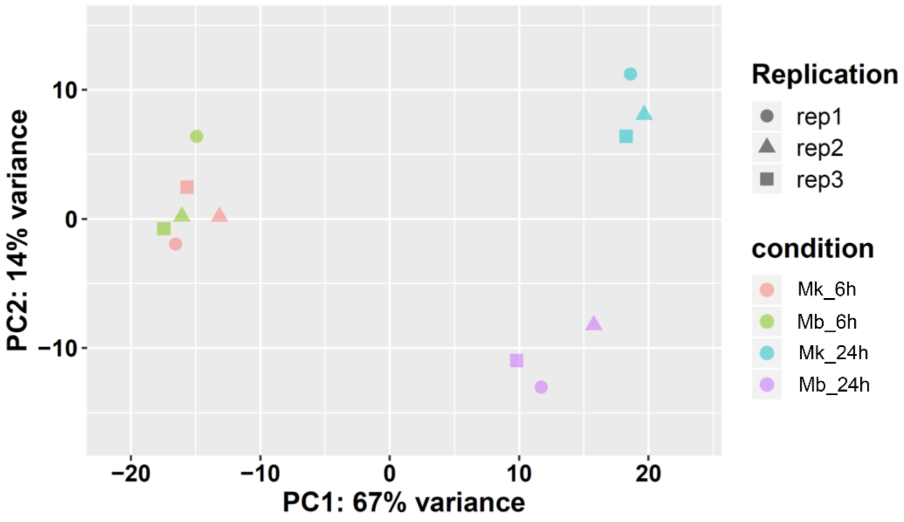

Supplementary Figure 1. Principal component analysis of transcriptome comparison

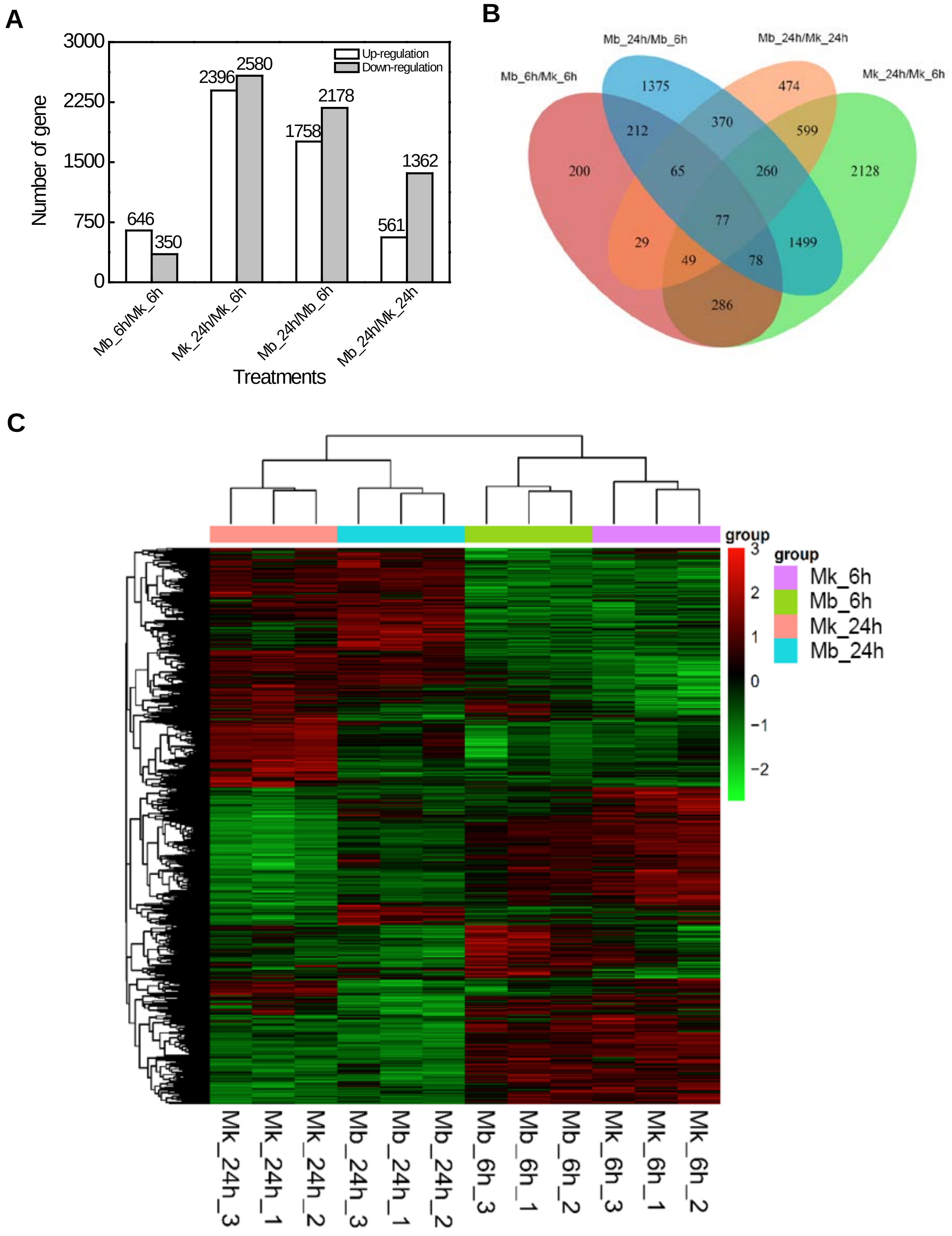

Supplementary Figure 2. Analysis of differential gene expression in *M. brunnea*-infected poplar leaves. (A) The upregulated and downregulated ($\log_2$ foldchange >1 and P <0.05) amount, **(B)** the Venn diagram and **(C)** heatmap and cluster analysis of expression level of differentially expressed genes (DEGs). Mk_6h_1/2/3 or Mk_24h_1/2/3, miRNA of one replicate from mock-treated poplar leaves at 6 or 24 hpi; Mb_6h_1/2/3 or Mb_24h_1/2/3, miRNA of one replicate from *M. brunnea*-inoculated poplar leaves at 6 or 24 hpi.

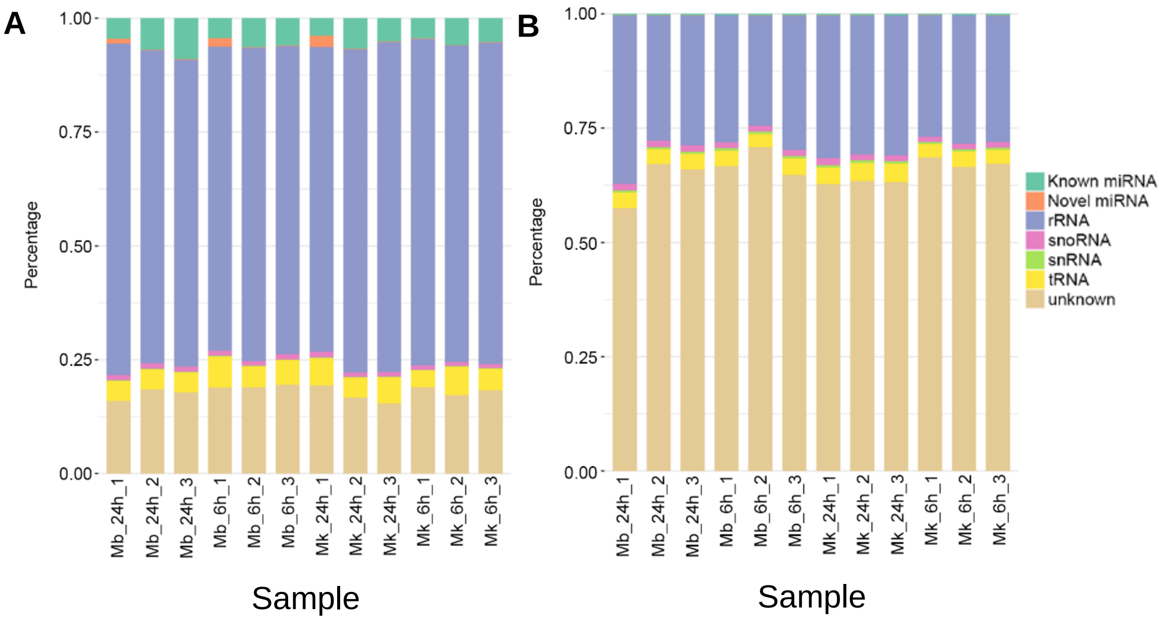

Supplementary Figure 3. MiRNAs composition results statistics. (A) total sequences; **(B)** unique sequences.

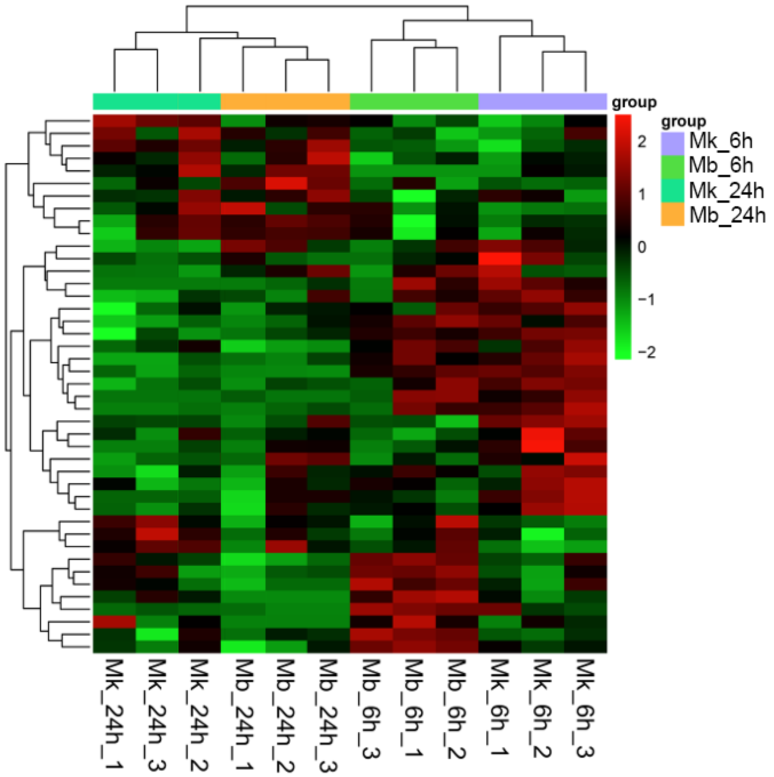

Supplementary Figure 4. Heatmap and cluster analysis of expression level of differentially expressed miRNAs (DEMs). Complete hierarchical clustering carried out using Euclidean distance, color coding was done according to the scale given. Green indicates downregulation and red upregulation.

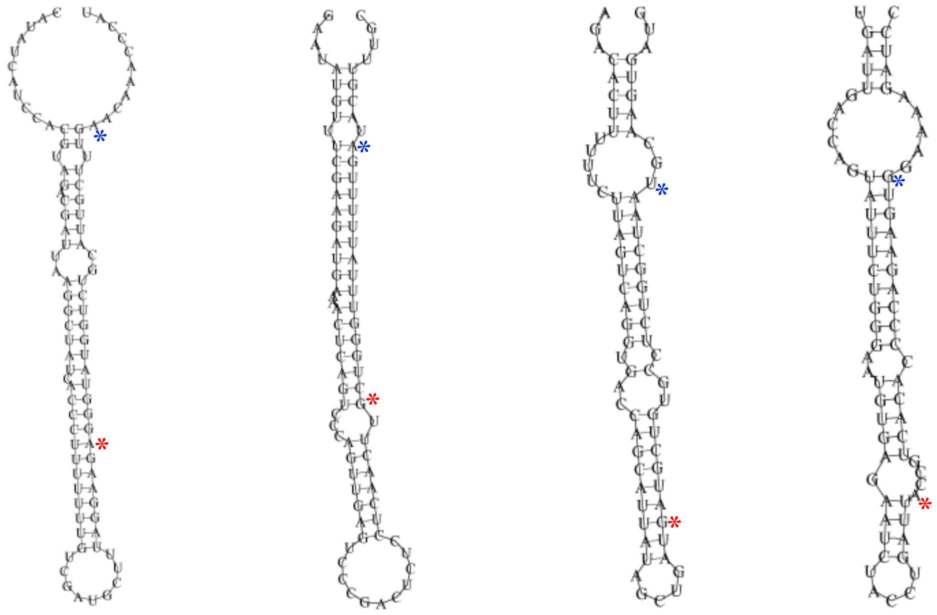

Novel MIR8567 Novel MIR3228 Novel MIR5913 Novel MIR8567

Supplementary Figure 5. The secondary structures of precursors of candidate novel miRNAs. Red and blue asterisks indicate the 5'- and 3'-end positions of mature novel miRNA on the secondary structure of precursor, respectively.
